# Safety of Zein Nanoparticles on Human Innate Immunity and Inflammation

**DOI:** 10.3390/ijms252111630

**Published:** 2024-10-29

**Authors:** Annunziata Corteggio, Tommaso Heinzl, Diana Boraschi, Silvia Voci, Agnese Gagliardi, Donato Cosco, Paola Italiani

**Affiliations:** 1 Institute of Biochemistry and Cell Biology (IBBC), National Research Council (CNR), 80131 Napoli, Italy; annunziata.corteggio@ibbc.cnr.it (A.C.); tommaso.heinzl@ibbc.cnr.it (T.H.); diana.boraschi@gmail.com (D.B.); 2 Shenzhen Institute of Advanced Technology (SIAT), Chinese Academy of Sciences (CAS), Shenzhen University of Advanced Technology, Shenzhen 518055, China; 3 China-Italy Joint Laboratory of Pharmacobiotechnology for Medical Immunomodulation (SIAT, CNR), Shenzhen 518055, China; 4 Stazione Zoologica Anton Dohrn (SZN), 80121 Napoli, Italy; 5 Department of Health Sciences, University “Magna Græcia” of Catanzaro, Campus Universitario “Salvatore Venuta”, 88100 Catanzaro, Italy; silvia.voci@unicz.it (S.V.); gagliardi@unicz.it (A.G.)

**Keywords:** innate immunity, zein, nanoparticles, innate memory, macrophage polarization

## Abstract

In recent years, natural polymers have attracted great interest for the development of release systems for vaccine formulations and drug delivery. Zein, a hydrophobic proline-rich protein mixture obtained from maize, is one of the most widely used polymers, very promising for applications in tissue engineering and the parenteral delivery of bioactive agents. Still, we have a limited understanding of the interaction between zein particles and the human immune system, in particular innate immunity/inflammation, which is the first line of defense of our body. Assessing the immune safety of nanoparticles is of central importance for ensuring that nano-formulations for medical use do not cause adverse effects on human health. Here, we evaluated the capacity of zein nanoparticles to induce/modulate the innate/inflammatory response, the development of innate memory, and the macrophage polarization by using reliable in vitro systems based on human primary monocytes and monocyte-derived macrophages. We observed that zein nanoparticles do not influence any of these aspects of the innate immune/inflammatory response, suggesting its safety and its potential efficiency as a nanocarrier for drug or antigen delivery.

## 1. Introduction

One of the main successes of the nanotechnologies is the development of nano-based drug delivery systems. The use of nanoparticles (NPs) has allowed us to overcome the limitations of free therapeutics, facilitate the crossing of biological barriers and the entry into cells, and, recently, the development of precision medicine [1] and of new vaccines, such as those for COVID-19 [2].

Once inside the body, NPs readily come in contact with the immune system, and, in particular, with the innate immune cells, the first line of immune defense against foreign agents and particles. Such interaction can lead to different outcomes, depending on whether NPs are recognized as threats or not. In the first case, an inflammatory reaction is triggered, while in the second case NPs are silently eliminated (either digested or excreted). Silent elimination encompasses excretion through the kidneys, mainly but not exclusively for colloidal systems below 5 nm [3], and non-inflammatory uptake and digestion by the mononuclear phagocyte system, which depends on the size and chemical composition of the particles [4].

Thus, one of the main concerns when new nanomaterial-based therapeutics are developed is to establish their potential immunotoxicity and capacity to activate unwanted innate/inflammatory immune responses [5]. The evaluation of immunotoxicity is especially important in the case of drug delivery for two main reasons: 1. to avoid tissue damage due to a persistent particle-induced inflammatory response; 2. to avoid recognition and elimination of the nano-drugs by the innate immune system and the consequent abolishment of their therapeutic function [6]. The evaluation of the innate immune activation capacity is important especially in case of nano-vaccination, in order to facilitate antigen presentation and the initiation/amplification of adaptive immunity against a tumor or infection in a controlled fashion [7].

Natural polymers have attracted great interest for the development of release systems for active agents, and zein is one of the most widely used [8]. Zein is a hydrophobic proline-rich protein mixture obtained from maize and is an ideal material in drug delivery studies, as it is generally regarded as safe (GRAS), has a favorable profile of biocompatibility, biodegradability and low toxicity, and originates from a renewable source [9]. Zein can form low-cost biodegradable flexible films and resistant hydrophobic coatings, which provide protection against microbial attack, suggesting its suitability for producing micro/nanoparticles to be used as delivery systems for nutrients and drugs [10]. The structure of the protein, rich in nonpolar amino acid residues, is promoting its use as a component of future innovative pharmaceutical formulations, especially nanoparticulate drug delivery systems, aiming at modulating the therapeutic efficacy and pharmacokinetic profiles of the entrapped compounds [11,12]. The yellow zein (about 90% pure) contains 8–9% xanthophyll pigments, such as lutein, zeaxanthin, and β-cryptoxanthin, which increase the inherent antioxidant properties of zein, favoring its scavenging activity against free radicals and lipid peroxidation [13].

Despite the wide range of possible applications of zein in tissue engineering and the parenteral delivery of bioactive agents [10,14,15], only a few studies have assessed the immune activation induced by zein-based carrier systems [16,17]. In these studies, however, it was shown that zein micro- and nanoparticles can be immunogenic. For example, Hurtado-López and Murdan reported that intramuscular injection of the zein microspheres induced the production of anti-zein IgG, whereas oral administration induced a mucosal IgA (but not IgG) anti-zein response [17]. These data were also confirmed by Li and co-workers, who demonstrated that the administration of zein nanoparticles to mice by intramuscular or subcutaneous routes induces high levels of specific IgG antibodies in the circulation, independent of the NP size [16]. Besides these few reports pertaining to zein immunogenicity and triggering of adaptive immune responses, our understanding of the interaction between zein particles and the immune system is still largely incomplete. In particular, there is no information on their interaction with the innate immune system.

Besides a direct activation in response to microbial and other challenges, innate immune cells have the capacity of developing the so-called “innate memory”, which allows the pre-exposed (primed) cells to react differently to a subsequent challenge [18]. The secondary response of primed cells can be stronger (potentiation, trained immunity) or weaker (tolerance) compared with the first response, in order to obtain better protection and less side effects upon repeated challenges. Circulating peripheral blood monocytes are recruited to tissues, where they differentiate in macrophages, which are professional phagocytes and antigen-presenting cells. According to their functions, newly incoming monocytes and tissue macrophages can be broadly classified into inflammatory (classically activated, M1) and anti-inflammatory (healing, M2) subsets, which play distinct roles in the initiation and resolution of inflammation [19,20]. Polarization of human monocytes/macrophages into functionally different subsets occurs in response to endogenous and exogenous stimuli and varies depending on the changes in the microenvironmental conditions (with M1 cells becoming M2 and vice versa) [19,20]. In the presence of microbial agents and inflammatory cytokines, monocytes/macrophages polarize toward the M1 functional phenotype and release reactive oxygen and nitrogen species and inflammatory cytokines to eliminate the dangerous agents [21]. In contrast, healing M2 cells control and resolve inflammation by releasing anti-inflammatory cytokines and take part in tissue healing and remodeling [22].

In this study, we aimed to investigate the effects on the human innate immunity of zein stabilized with sodium cholate. Several works have already described the contribution of sodium cholate and its derivatives in the formation of stable colloidal NPs [23,24,25]. In fact, the use of specific surfactants in the development of novel formulations for (nano)vaccination can be profitable considering their inherent adjuvant properties [26] and their characteristics of absorption enhancers [27]. Moreover, these features can be exploited in order to enhance the uptake of the encapsulated antigen by the immune cells [28,29].

We have used human primary monocytes in vitro as a reliable model for assessing human innate immunity. We have evaluated the capacity of zein NPs to induce a primary innate/inflammatory response (measured as the balance between the induction of inflammatory and anti-inflammatory cytokines) and also their innate memory-inducing capacity. In addition, we have generated macrophages from blood monocytes, using an established in vitro differentiation system, and assessed the capacity of zein NPs to modulate the M1 and M2 macrophage polarization.

## 2. Results and Discussion

### 2.1. Physical–Chemical Characteristics of Zein NPs

The lack of knowledge of in vivo behavior of zein NPs, once systemically administered, requires the investigation of their stability in biological fluids.

**Figure 1:**
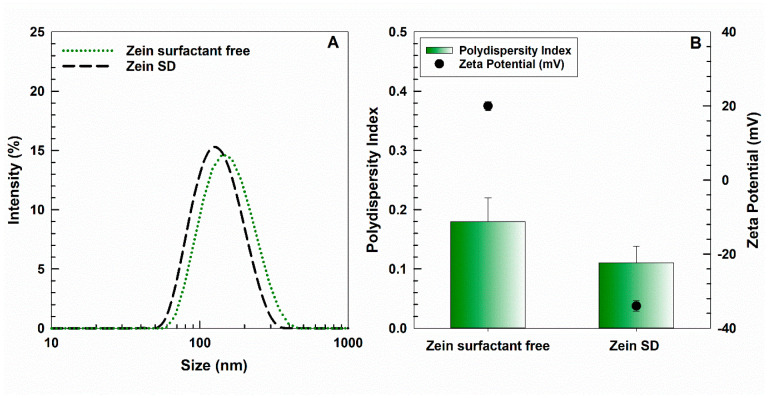
(**A**) Mean sizes, (**B**) polydispersity index, and zeta potential of surfactant-free and SD-stabilized zein NPs (2 mg protein/mL water). The analysis was performed in MilliQ water (dilution 1:50). The conductivity of samples was ~0.003 mS/cm.

We recently developed zein NPs and investigated their physical–chemical characteristics in colloidal formulations in order to assess their suitability as drug carriers [30]. As shown in Figure 1A, the hydrodynamic diameter of the surfactant-free zein NPs in water is ~160 nm, with a polydispersity index (PdI) of ~0.2 and a positive zeta potential. The addition of the anionic surfactant sodium deoxycholate (SD, 1.25% *w*/*v*) caused a significant decrease in the PdI (~0.1) and affected the zeta potential of the NPs by shifting this parameter from positive to negative values, likely due to different exposures to the protein’s charged residues [31]. In previous studies, investigation of the NP morphology via Transmission Electron Microscopy demonstrated a spherical shape of both surfactant-free and SD-coated zein NPs and confirmed the data of Dynamic Light Scattering [30,31].

**Figure 2:**
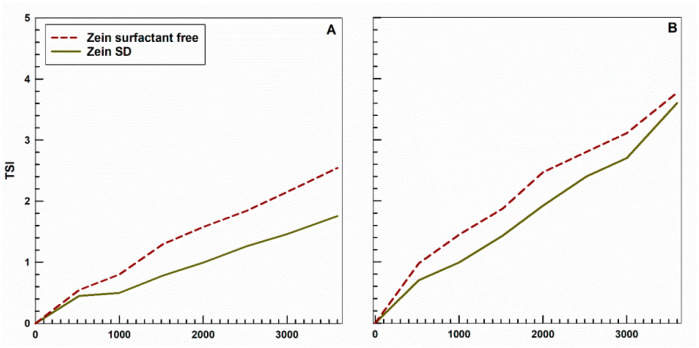
Turbiscan stability index (TSI) profile of surfactant-free and SD-stabilized zein NPs (2 mg protein/mL water). (**A**): 25 °C; (**B**): 37 °C.

The stability profiles of the NPs were investigated using the Turbiscan Lab Expert^®^, an analytical tool capable of detecting differences in particle sizing (aggregation/flocculation) or phase separation behavior (creaming/sedimentation). In particular, TSI curves were used to compare the two zein NP preparations as a function of the time and temperature. As shown in Figure 2, both formulations showed excellent stability at 25 °C. A temperature-dependent increase in TSI slopes was observed, but to a limited extent (values of less than 4), overall suggesting a good stability of the colloidal systems (Figure 2).

**Figure 3:**
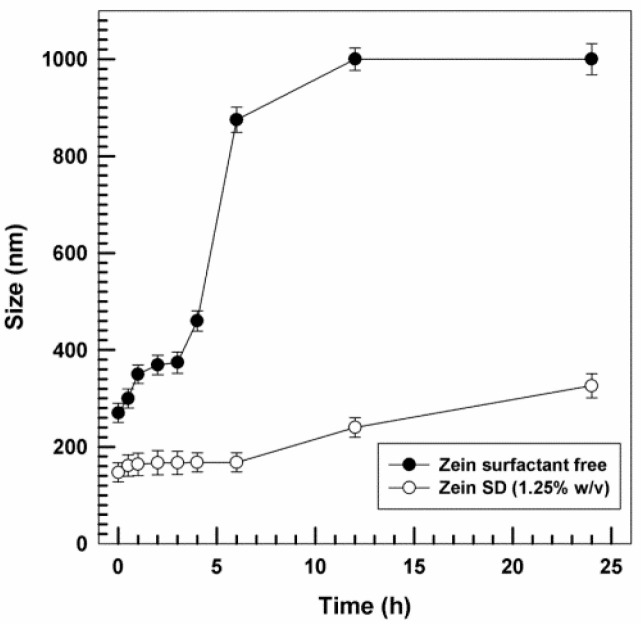
Stability of zein NPs in human serum. Zein NPs (2 mg/mL) were incubated in 70% heat-inactivated human AB serum at 37 °C for increasing times. Both naked NPs and surfactant-stabilized NPs were used. Data are the mean ± sd of triplicate evaluations.

In view of a future use in vivo, the stability of zein NPs in biological fluids was examined. It was previously reported that zein NPs stabilized with SD incubated with 70% FBS at 37 °C maintain their average size (≤200 nm) up to 24 h, while the surfactant-free NPs showed a constant increase in their mean diameters [30]. Here, surfactant-free and SD-coated zein NPs were incubated in human serum. In line with previous results [30], no substantial changes in their mean diameters were observed for SD-stabilized zein NPs up to 24 h of incubation with human serum at 37 °C, while the surfactant-free NPs showed a massive size increase (Figure 3).

These data suggest that the SD-coated zein NPs are stable in a human biological context and were selected for assessing their interaction with human immune cells.

### 2.2. Primary Innate Response of Human Monocytes Exposed to Zein NPs

The innate immune system is responsible for the early defense toward infection and disease and includes a widely distributed network of cells that “patrol” the body. Among the innate immune cells, circulating monocytes and tissue-resident macrophages are key players in the initiation, development, and resolution of an inflammatory response. The specific interaction of monocytes and macrophages with NPs may hold great promise for the application of nanotechnology in biomedicine, for drug or vaccine delivery, immunomodulation, or specific cell targeting. Human monocytes and macrophages react to microbial and other stimuli by mounting a potent inflammatory response, aiming at destroying the potential danger. Notably, cells that have been pre-exposed to activating agents can react to a subsequent challenge in a way that differs from the primary response. This phenomenon is recognized as innate immune memory. We aimed to determine whether the surfactant-coated zein NPs can induce or modulate a primary inflammatory response in human monocytes and induce or interfere with the generation of innate memory. The primary response of monocytes to zein NPs (100 ng/mL) was evaluated after exposure in culture medium for 24 h. Zein NPs were pre-incubated with heat-inactivated human AB serum (see Materials and Methods) in order to mimic the formation of the biocorona, a process that occurs when NPs are introduced into a biological environment such as blood [32]. The NP concentration was selected as the highest endotoxin-free concentration from dose–response experiments and roughly corresponds to an intravenous bolus administration of 0.1 mg NPs/kg body weight.

**Figure 4:**
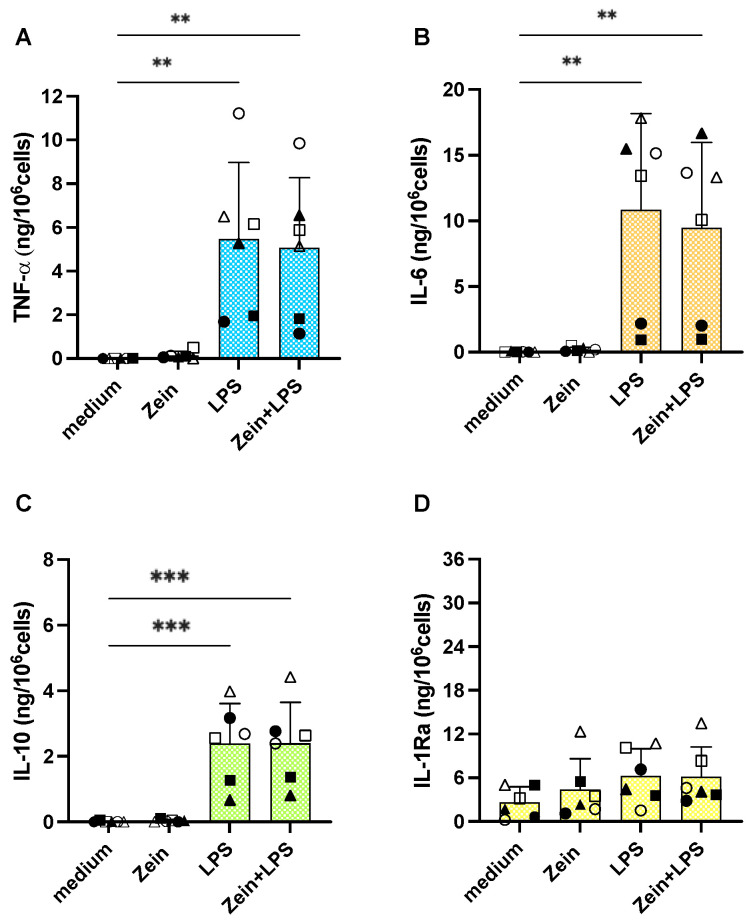
Primary innate immune response of human monocytes to zein NPs, LPS, or their mixture. Human blood monocytes were exposed in culture to medium alone or containing zein NPs (100 ng/mL), LPS (1 ng/mL), or zein NPs + LPS. The levels of inflammatory (TNFα, IL-6; panels (**A**,**B**)) and anti-inflammatory cytokines (IL-10, IL-1Ra; panels (**C**,**D**)) were measured in the 24 h supernatants via ELISA. The columns represent the average value + sd from 6 individual donors. The individual values are indicated with different symbols. Statistical significance: *** *p* < 0.001 and ** *p* < 0.01 in the comparisons between medium vs. LPS and NPs + LPS. All other comparisons (medium vs. NPs, LPS vs. NPs + LPS) are not significant.

Freshly isolated human blood monocytes were exposed in vitro to culture medium alone or containing serum-coated zein NPs, the bacterial agent LPS (1 ng/mL; from *E. coli*) as positive control (LPS is an excellent activator of human monocyte innate/inflammatory responses), or the mixture of LPS with the zein NPs. Monocyte activation was evaluated in terms of the production and release of four cytokines, the inflammatory factors TNFα and IL-6, and the anti-inflammatory cytokines IL-10 and IL-1Ra, and the inflammatory chemokine IL-8 (Figure S2A). Results in Figure 4 show that zein NPs do not induce a measurable reactivity in human monocytes, being unable to induce the two inflammatory cytokines (Figure 4A,B). LPS significantly activated the production of inflammatory factors in monocytes, although with a substantial donor-to-donor variability. Zein NPs did not affect the reactivity of the monocytes to LPS (Figure 4A,B). When examining the production of anti-inflammatory cytokines, LPS stimulation induced the production of IL-10 but did not significantly increase the measurable basal levels of IL-1Ra, although again with some donor-to donor variability (Figure 4C,D). Also, in the case of anti-inflammatory factors, zein NPs did not show a direct activation effect, nor did they modulate the LPS-induced activation (Figure 4C,D).

### 2.3. Innate Memory Response of Human Monocytes Exposed to Zein NPs

**Figure 5:**
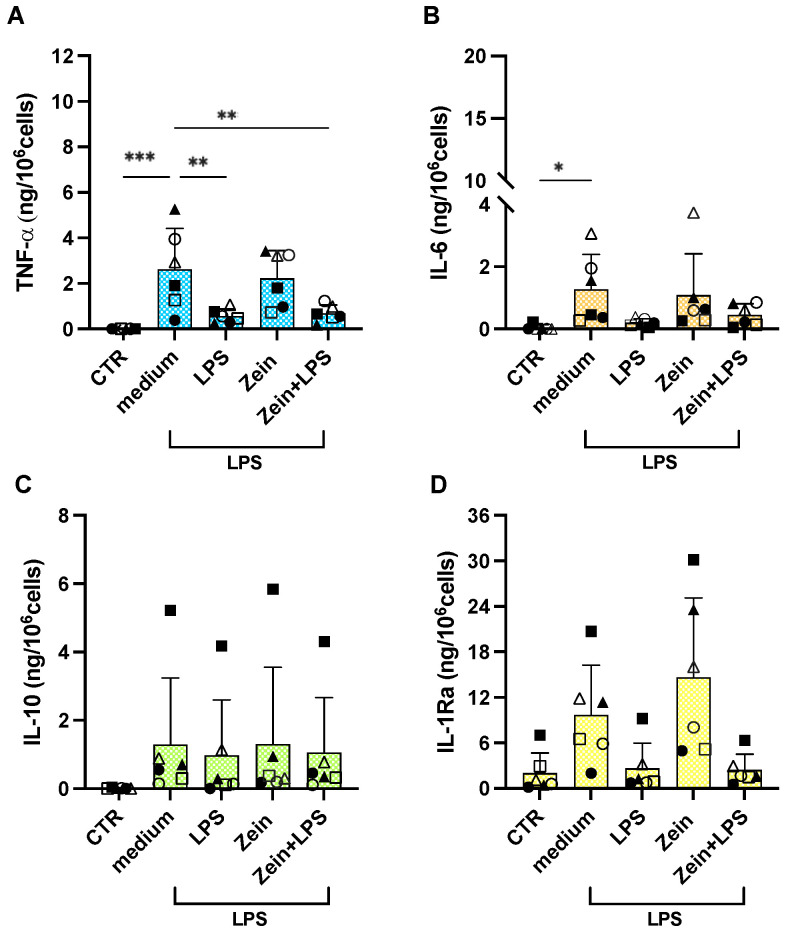
Secondary “memory” response of human monocytes primed with LPS, zein NPs, or their mixture. After a primary exposure to medium alone, LPS (1 ng/mL), zein NPs, or NPs admixed with LPS (see Figure 2), cells were washed and rested in culture for 7 days to allow extinction of the primary activation, then challenged for 24 h with medium or with LPS (5 ng/mL). Controls (CTR columns) include cells primed with medium, NPs, LPS, or LPS + NPs, which were all at baseline after the challenge with medium alone, thereby confirming the complete extinction of the primary activation. Inflammatory (TNFα, IL-6; panels (**A**,**B**)) and anti-inflammatory cytokines (IL-10, IL-1Ra; panels (**C**,**D**)) were measured in the 24 h supernatants via ELISA. The columns represent the average value + sd from 6 individual donors. The individual values are indicated with different symbols. Statistical significance: * *p* < 0.05 unchallenged controls CTR vs. medium-primed LPS-challenged (medium) for IL-6; ** *p* < 0.01 medium-primed LPS-challenged (medium) vs. LPS-primed LPS-challenged (LPS) and vs. Zein+LPS-primed LPS-challenged for TNFα; *** *p* < 0.001 unchallenged controls CTR vs. medium-primed LPS-challenged (medium) for TNFα. Other comparisons (CTR vs. medium for IL-1Ra, medium vs. LPS for IL-10 and IL-1Ra, medium vs. Zein, LPS vs. Zein + LPS) are not significant.

After exposure for 24 h to zein NPs, LPS, or their mixture, cells were washed (to eliminate the priming agents) and rested for 7 days to allow cells to return to baseline conditions. It should be noted that after 7 days in culture, monocytes spontaneously differentiate into macrophages. The extinction of cell activation was confirmed by examining the production of cytokines released in the culture medium at the end of the resting period and by the fact that no cytokines were produced in primed cells upon a further incubation for 24 h in medium alone (Figure 5). After the resting period, cells were either exposed to medium alone (groups CTR in Figure 5) or challenged with LPS (5 ng/mL). A higher LPS concentration at challenge is meant to represent a more severe infectious event [18].

As for the primary response, the memory response was evaluated in terms of the production of inflammatory and anti-inflammatory cytokines. LPS challenge of medium-primed cells showed a general induction of TNFα, IL-6, IL-10, and IL-1Ra production, and the inflammatory chemokine IL-8 (Figure S2B). Notably, despite a five times higher LPS challenge, the production of inflammatory cytokines by unprimed (medium-primed) macrophages was much lower than that of fresh monocytes (compare Figure 4A,B with Figure 5A,B). This underlines the fact that the major inflammatory effector cells are the newly recruited blood monocytes, whereas tissue macrophages are much less reactive to stimuli (to better preserve tissue integrity). As expected, priming with LPS generated a tolerance type of memory response relative to inflammatory cytokines, i.e., a lower production of TNFα and IL-6 in response to an LPS challenge as compared with medium-primed control cells (Figure 5A,B), although the reduction in IL-6 was evident but not statistically significant (because of donor-to-donor variability). On the other hand, LPS priming did not seem to induce a lower responsiveness in terms of anti-inflammatory cytokines (Figure 5C,D). This is in line with the notion that a secondary response to a substantial bacterial challenge down-regulates the production of inflammatory factors to a level that prevents severe tissue damage, while maintaining efficient anti-inflammatory machinery to control the extent and duration of the defensive reaction [33,34].

The presence of zein NPs appears inconsequential also on innate memory generation. Priming with zein NPs alone did not induce a memory response to LPS, as shown by the same response to an LPS challenge by medium-primed and NP-primed cells, for both inflammatory and anti-inflammatory cytokines (Figure 5A–D). Likewise, the memory response induced by LPS priming (decrease in inflammatory cytokines and unchanged levels of anti-inflammatory cytokines upon challenge) was not affected if cells had been primed with LPS admixed with zein NPs (Figure 5A–D).

Despite some donor-to-donor variability, the overall result is that zein NPs appear immunologically safe, i.e., unable to trigger an innate/inflammatory response or to generate/modulate innate memory, in human primary monocytes/macrophages.

### 2.4. Effects of Zein NPs on M1 and M2 Macrophage Polarization

**Figure 6:**
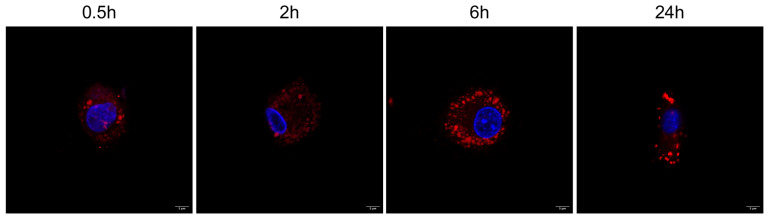
In vitro uptake of rhodamine-labeled zein (red) in monocyte-derived macrophages. Cell nuclei are stained blue with Hoechst 33258. Representative images for 0.5, 2, 6, and 24 h time points are shown. Bar 5 μm.

We first evaluated the uptake of zein NPs by monocyte-derived macrophages. Cells were incubated with rhodamine labeled-zein NPs (25 ng/mL) for different periods of time (0.5, 2, 6, 24 h), in order to assess the cell-entry kinetics and persistence of zein inside the cells. Fluorescence microscopy analysis showed that zein NPs were rapidly internalized by macrophages (0.5 h) and persisted within cells throughout the entire experimental time (Figure 6).

We then examined the ability of zein NPs to affect the capacity of macrophages to adopt different functional phenotypes in response to microenvironmental cues by studying the polarization of human monocyte-derived macrophages into the M1 functional phenotype (classically activated inflammatory activation) and the M2 healing phenotype (anti-inflammatory tissue-remodeling activation). Naïve macrophages were obtained by culturing freshly isolated human blood monocytes with the growth/differentiation factor CSF-1 for 7 days. These cells were then polarized into the M1 and M2 functional phenotypes by exposure for 24 h to LPS + IFN-γ and IL-10, respectively, in the absence or presence of zein NPs. Differentiation was evaluated in terms of the expression of surface markers specific to mature macrophages such as CD80 and 25F9, highly expressed in naïve M1 and M2, and CD206, mainly expressed in M2 (Figure S1).

**Figure 7:**
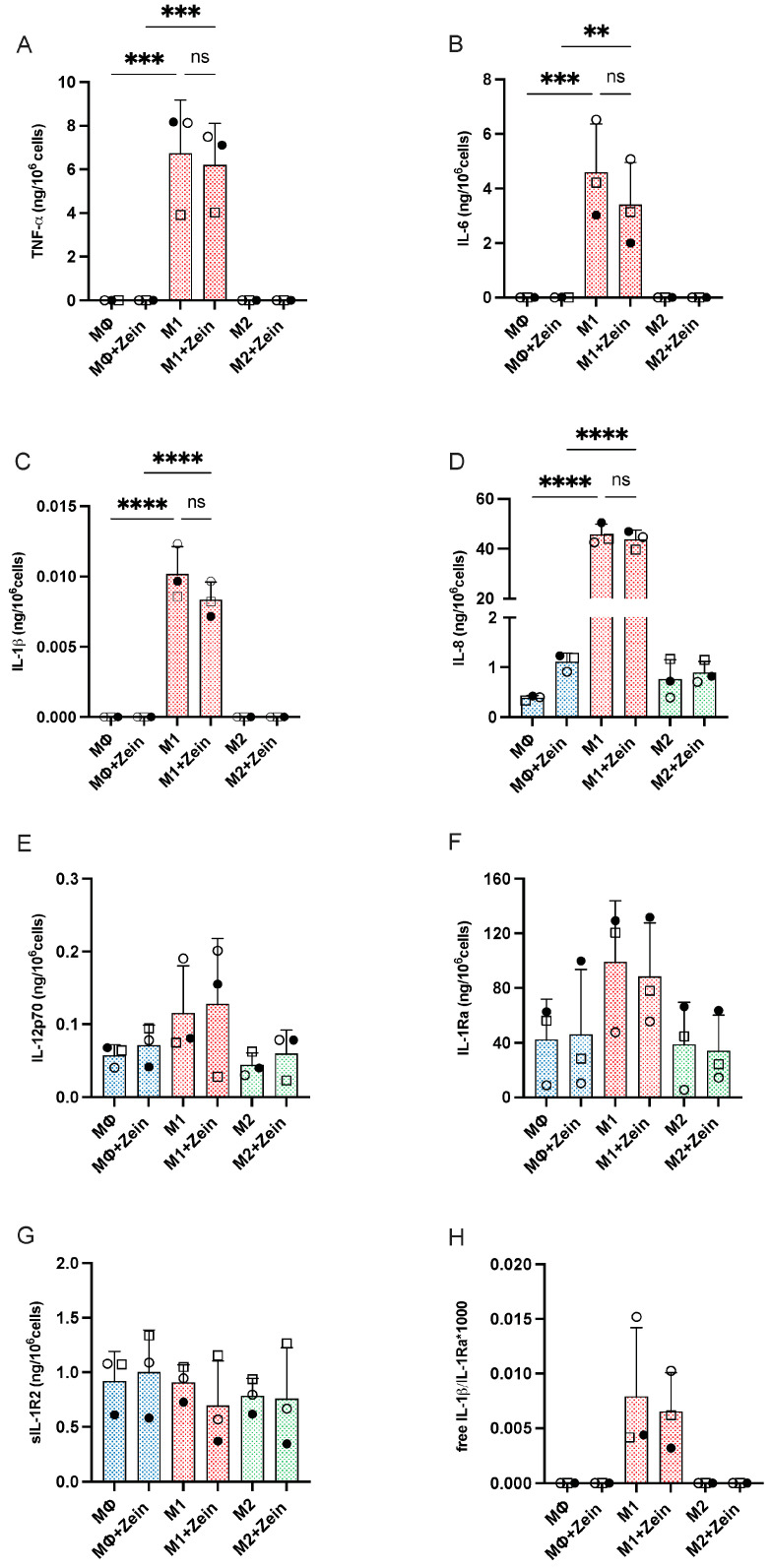
Effect of zein NPs on human macrophage polarization. Monocyte-derived macrophages were either left untreated (naïve, 24 h in culture medium alone—MΦ) or polarized toward the M1 or the M2 functional phenotypes (by 24 h exposure to LPS + IFN-γ for M1, and IL-10 for M2) in the absence or in the presence of 100 ng/mL zein NPs. The production of cytokines and other inflammation-related factors was measured via ELISA in the 24 h supernatants: (**A**), TNFα; (**B**), IL-6; (**C**), IL-1β; (**D**), IL-8; (**E**), IL-12p40; (**F**), IL-1Ra; (**G**), sIL-1R2. Panel (**H**) reports the calculated free active IL-1β, i.e., the ratio between free IL-1β (the fraction of the cytokine not bound by its inhibitory receptor sIL-1R2) and the receptor antagonist IL-1Ra multiplied by 1000. The columns represent the average value + sd from 3 individual donors. Individual values are shown by different symbols. Statistical significance: ** *p* < 0.01 MΦ + Zein vs. M1 + Zein for IL-6; *** *p* < 0.001 MΦ vs. M1 for TNFα and IL-6. MΦ + Zein vs. M1 + Zein for TNFα; **** *p* < 0.0001 MΦ vs. M1 or MΦ + Zein vs. M1+Zein for IL-8 and IL-1β. Other comparisons (M1 or M2 vs. M1 + NP or M2 + NP, MΦ vs. M2) are not significant.

Polarization was functionally evaluated in terms of the release of different cytokines, i.e., the inflammatory factors TNF-α, IL-6, and IL-1β, the T cell-activating cytokine IL-12p70, the chemokine IL-8, and the anti-inflammatory factors IL-1Ra and sIL-1R2 (both inhibitors of IL-1). Results in Figure 7 show that cells polarized into M1 produce significant levels of the inflammatory cytokines (TNF-α IL-6, IL-1β) and of the chemokine IL-8, whereas production of IL-12 and of the anti-inflammatory factors was not increased over the basal levels. The M2 macrophages did not produce inflammatory factors, and, similar to M1 cells, their production of IL-12 and anti-inflammatory factors was not different from basal levels. The presence of zein NPs during the polarization phase did not change the polarization profile (Figure 7), although some donor-to-donor variability was observed.

## 3. Materials and Methods

### 3.1. Preparation of Zein NPs

Yellow zein (CAS number 9010-66-62, MW 22–24 KDa) and sodium deoxycholate monohydrate (SD) were purchased from Merck Sigma-Aldrich^®^ (Darmstadt, Germany).

Zein nanoparticles were obtained using the nanoprecipitation technique as previously reported [35]. Briefly, zein (10 mg/mL) in 3 mL of an ethanol/aqueous solution (2:1 *v*/*v*) was added with 5 mL MilliQ water alone or containing 12.5 mg/mL of the anionic surfactant SD at room temperature. The obtained suspension was homogenized for 1 min with an Ultraturrax^®^ (model T25, IKA^®^ Werke, Staufen, Germany) at 24,000 rpm and then mechanically stirred at 600 rpm for 12 h to promote the evaporation of the organic solvent. The final zein concentration was 2 mg/mL. Subsequently, the NPs were purified using Amicon^®^ Ultra centrifugal filters, Sigma-Aldrich (cut-off 10 kDa, 4000 rpm for 120 min) for the in vitro experiments. NPs were resuspended in different media, based on the experiments to be performed [36].

### 3.2. Physical–Chemical Characterization of Zein NPs

The physical–chemical parameters of NPs such as the mean diameter, size distribution, and zeta potential were investigated via photon correlation spectroscopy (Zetasizer NanoZS, Malvern Panalytical Ltd., Spectris plc, Great Marvern, UK) with an applied third order cumulant fitting correlation function. The results are expressed as a function of the intensity parameter and are the means of three different measurements carried out in triplicate (10 determinations for each replicate) on three different samples ± standard deviation.

The kinetic stability of the formulations was investigated using the Turbiscan Lab Expert^®^ analyzer (Formulaction, Toulouse, France) as a function of temperature and incubation time, as previously described [35]. The data were processed by a Turby Soft 2.0 and reported as the Turbiscan Stability Index (TSI) versus time.

To assess the behavior of zein NPs in the presence of biological fluids, zein NPs were exposed to human serum at 37 °C. Briefly, 20 µL of purified NPs (400 μg of protein) was added to 1 mL of 70% AB serum heat-inactivated, and the resulting suspension was incubated at 37 °C for 24 h under stirring. The average diameter of the samples was analyzed at different incubation times as described [37].

### 3.3. Biocorona Formation on Zein NPs

Before addition to cell culture, zein NPs were pre-incubated in 50% heat-inactivated human AB serum (Sigma-Aldrich) at 37 °C for 1 h under stirring in order to obtain the formation of a serum biocorona on the surface, thereby ensuring particle stability in culture. The serum–NPs mixture was then added directly to culture wells, adjusting NP and serum concentration to the desired values for each treatment.

### 3.4. Human Monocyte Isolation

Buffy coats collected in bags with sodium citrate as anticoagulant were obtained from six healthy donors, upon informed consent. The procedure was in agreement with the Declaration of Helsinki, and the protocol was approved by the Regional Ethics Committee for Clinical Experimentation of the Tuscany Region (Ethics Committee Register n. 14,914 of 16 May 2019).

Monocytes were isolated by CD14 positive selection with magnetic microbeads (Miltenyi Biotec, Bergisch Gladbach, Germany) from peripheral blood mononuclear cells (PBMC), obtained via Ficoll-Paque gradient density separation (GE Healthcare, Bio-Sciences AB, Uppsala, Sweden). Monocyte preparations used in the experiments were > 95% viable and >95% pure (assessed by trypan blue exclusion and cytosmears).

Monocytes (5 × 10^5^ cells/well) were cultured in RPMI-1640 medium + Glutamax™ (GIBCO™, ThermoFisher Scientific, Waltham, MA, USA) supplemented with 50 μg/mL gentamicin sulfate (GIBCO), 5% heat-inactivated human AB serum (Merck Sigma-Aldrich^®^), and 10 ng/mL CSF-1 (Merck Sigma-Aldrich^®^) in a final volume of 1.0 mL in wells of 24-well flat bottom plates (Corning Costar, Corning Inc. Life Sciences, Oneonta, NY, USA). The cells are maintained at 37 °C in a 5% CO_2_ atmosphere. Monocyte stimulation was performed after overnight resting.

### 3.5. Human Monocyte Activation and Induction of Innate Memory

For assessing the primary innate/inflammatory response, monocytes were exposed for 24 h to culture medium alone (medium/negative control) or containing 1 ng/mL LPS (positive control; from *E. coli* O55:B5; Merck Sigma-Aldrich^®^), serum pre-coated zein NPs, and the mixture LPS + zein NPs.

For the memory experiments, after the first exposure to stimuli for 24 h and supernatant collection, cells were washed and cultured for 7 additional days, the medium refreshed every 3 days. During this period (resting phase), the activation induced by previous stimulation subsided, and cells returned to their baseline status. This was previously determined by the lack of production/release of inflammation-related cytokines (mRNA and proteins) in time course experiments. After the resting phase, the supernatant was replaced with fresh medium alone or containing 5 ng/mL LPS, and incubation was carried out for 24 h (challenge).

All supernatants (after the first stimulation, after the resting phase, and after challenge) were frozen at −80 °C for subsequent cytokine analysis.

Cell viability was monitored during the entire course of culture by visual inspection (viable cell counting in phase contrast optical microscopy). No visible changes in the cell number and viability were identified in response to the different treatments. Duplicate samples were prepared for each experimental condition.

### 3.6. Human Monocyte-Derived Macrophages Activation and Polarization

Monocytes were cultured in complete culture in the presence of 10 ng/mL CSF-1 (Merck Sigma-Aldrich^®^) in a final volume of 1.0 mL in wells of 24-well flat bottom plates (Corning Costar). The cells were maintained at 37 °C in a 5% CO_2_ atmosphere for 7 days to allow spontaneous monocyte differentiation into resting naïve macrophages. The culture medium was refreshed every three days. On day 8, the supernatant was removed, and naïve macrophages were polarized toward M1 or M2 in the absence or presence of 100 ng/mL of zein NPs. M1 polarization was obtained by incubation for 24 h with LPS (10 ng/mL) and human recombinant IFN-γ (20 ng/mL; R&D Systems, Minneapolis, MN, USA). M2 polarization was obtained by adding human recombinant IL-10 (20 ng/mL; R&D Systems) for 24 h. Control naïve macrophages were incubated for 24 h in culture medium alone. Supernatants were collected and frozen at −80 °C for subsequent cytokine analysis.

Cell viability was monitored during the entire course of culture by visual inspection. No visible changes in the cell number and viability were identified in response to the different treatments. Duplicate samples were prepared for each experimental condition.

### 3.7. Analysis of Surface Markers on Monocyte-Derived Macrophages

Surface markers of naïve, M1, and M2 macrophages were assessed using an immunofluorescent antibody-labeling technique. At the end of the differentiation and polarization period, monocyte-derived macrophages were washed twice in PBS and processed as follows. For the immunofluorescent analysis of CD80 (a costimulatory protein expressed on professional antigen-presenting cells including macrophages), the samples were fixed with 4% paraformaldehyde for 15 min, permeabilized with 0.25% Triton X100/PBS, blocked with 2% BSA/PBS (30 min at RT), incubated with an anti-CD80 (B7-1) rabbit polyclonal antibody (cat. PA5-85913, Invitrogen, Waltham, MA, USA) diluted 1:100 (o/n at 4 °C), and then with a goat anti-rabbit IgG labeled with Alexa Fluor 568 (red) (30 min at RT). For the analysis of CD206 (a protein expressed on human macrophages and generally used as a marker for detecting M2 macrophages), the samples were fixed with 4% paraformaldehyde for 15 min, permeabilized with 0.1% Triton X-100/PBS, blocked with 10% serum/PBS (45 min at RT), incubated with an anti-CD206 rabbit polyclonal antibody (cat. PA5-101657, Invitrogen) diluted 1:200 (o/n at 4 °C), and then with a goat anti-rabbit IgG labeled with Alexa Fluor 568 (red) (30 min at RT). For the analysis of the Mature Macrophage Marker 25F9 (a protein present on mature macrophages both on the cell surface and in intracellular vesicular structures), the samples were fixed with acetone for 15 min at RT, blocked with 2% BSA/PBS (30 min at RT), incubated with an anti-Mature Macrophage Marker mouse monoclonal IgG antibody (eBio25F9 (25F9), cat. 14-0115-82 eBioscience, Invitrogen) diluted 1:100 (o/n at 4 °C), and then with a goat anti-mouse IgG labeled with Alexa Fluor 568 (red) (30 min at RT). For all the samples, nuclei were stained with Hoechst 33258 (blue). Eventually, cells were washed three times in PBS and once in sterile water to remove salts. Coverslips were then mounted on glass microscope slides with Mowiol. Immunolocalization of 25F9, CD80, and CD206 was visualized and images were obtained using a Zeiss LSM700 laser-scanning confocal microscope with a 63× oil-immersion objective (Zeiss, Jena, Germany).

### 3.8. Evaluation of Zein Uptake

Monocyte-derived macrophages, plated on glass coverslips at the density of 2 × 10^5^ cells/slide, were treated with rhodamine labeled-zein NPs (25 ng/mL) for different time periods (30′, 2 h, 6 h, 24 h). At the end of each experimental time, cells were washed twice in PBS, fixed with 4% paraformaldehyde for 10 min, incubated with Hoechst 33258 to label the nuclei, and washed three times in PBS and once in sterile water to remove salts. Coverslips were then mounted on glass microscope slides with Mowiol. Localization of zein NPs was visualized, and images were obtained using a Zeiss LSM700 laser-scanning confocal microscope with a 63× oil-immersion objective (Zeiss, Jena, Germany).

### 3.9. Evaluation of Cytokine Production

Production of cytokines was measured in the culture supernatants via ELISA (R&D Systems, Inc., Minneapolis, MN, USA) using a MultiScan FC reader (ThermoScientific, Waltham, MA, USA) according to the manufacturer’s instructions. Two ELISA replicates were run for each sample.

### 3.10. Statistical Analysis

Cytokine levels are presented as ng per million input monocytes. Graphical presentations and statistical analysis were performed using GraphPad Prism 9 (GraphPad Inc., La Jolla, CA, USA).

Data are shown as averages of biological duplicates or as averages of technical replicates of biological duplicates, and results are reported as mean + standard deviation (sd) of values. Statistical analysis was carried out using a one-way ANOVA unpaired with the Dunnett test for multiple comparisons. For polarization experiments, a one-way ANOVA unpaired with the Bonferroni test was used.

## 4. Conclusions

Zein NPs are promising nanocarriers for medical use, thanks to their favorable biocompatibility and physical–chemical properties [38]. Here, we aimed to investigate the immune safety of these NPs on human primary monocytes/macrophages in a representative in vitro model by assessing their capacity to induce or modulate 1. the innate/inflammatory response, 2. the development of innate memory, and 3. the macrophage polarization. In general, the evaluation of NP immunosafety is limited to toxicity on cell lines or in initiating pathology-related inflammation, while the evaluation of their effects on human primary cells and their capacity to interfere with the physiological course of the innate immune response is often neglected. We observed that the presence of zein NPs, at the size and concentrations used, did not influence the normal development of a defensive innate/inflammatory response to a bacterial challenge, suggesting that zein NPs do not affect the immunological fitness of the host. Likewise, zein NPs did not directly induce memory, i.e., did not influence the response of monocytes to subsequent stimuli, and did not drive the macrophage polarization toward a functional phenotype, again suggesting no interference with the normal development of innate immune responses. This makes us hypothesize that zein NPs are not perceived as a danger by the innate immune system and therefore do not trigger any inflammatory defensive reaction [39]. However, they seem to be retained within cells for a long period (at least 24 h) without being degraded, suggesting the possibility of a persistent delivery of therapeutic cargos. These observations make them an excellent candidate for future medical uses.

## Data Availability

Data supporting reported results can be found in the Supplementary Materials or are available from the authors upon request.

## Supplementary Materials

The following supporting information can be downloaded at: https://www.mdpi.com/article/10.3390/ijms252111630/s1.
