# Supplementary material for: Safety of Zein Nanoparticles on Human Innate Immunity and Inflammation
Source: Int J Mol Sci. 2024 Oct 29;25(21):11630. doi: 10.3390/ijms252111630 (PMC11546227; doi:10.3390/ijms252111630)
Supplement: Supplementary file 1 [file ijms-25-11630-s001.zip › ijms-3144112-supplementary.pdf]

Supplementary Materials

Safety of Zein Nanoparticles on Human Innate Immunity and Inflammation

Annunziata Corteggio [1], Tommaso Heinzl [1], Diana Boraschi [1,2,3,4], Silvia Voci [5], Agnese Gagliardi [5], Donato Cosco [5]* and Paola Italiani [1,3,4]*

[1] Institute of Biochemistry and Cell Biology (IBBC), National Research Council (CNR), 80131 Napoli, Italy; annunziata.corteggio@ibbc.cnr.it (A.C.); tommaso.heinzl@ibbc.cnr.it (T.H.); diana.boraschi@gmail.com (D.B.)

[2] Shenzhen Institute of Advanced Technology (SIAT), Chinese Academy of Sciences (CAS), Shenzhen University of Advanced Technology, Shenzhen 518055, China

[3] China-Italy Joint Laboratory of Pharmacobiotechnology for Medical Immunomodulation (SIAT, CNR), Shenzhen 518055, China

[4] Stazione Zoologica Anton Dohrn (SZN), 80121 Napoli, Italy

[5] Department of Health Sciences, University "Magna Græcia" of Catanzaro, Campus Universitario "Salvatore Venuta", 88100 Catanzaro, Italy; silvia.voci@unicz.it (S.V.); gagliardi@unicz.it (A.G.)

* Correspondence: donatocosco@unicz.it (D.C.); paola.italiani@ibbc.cnr.it (P.I.)

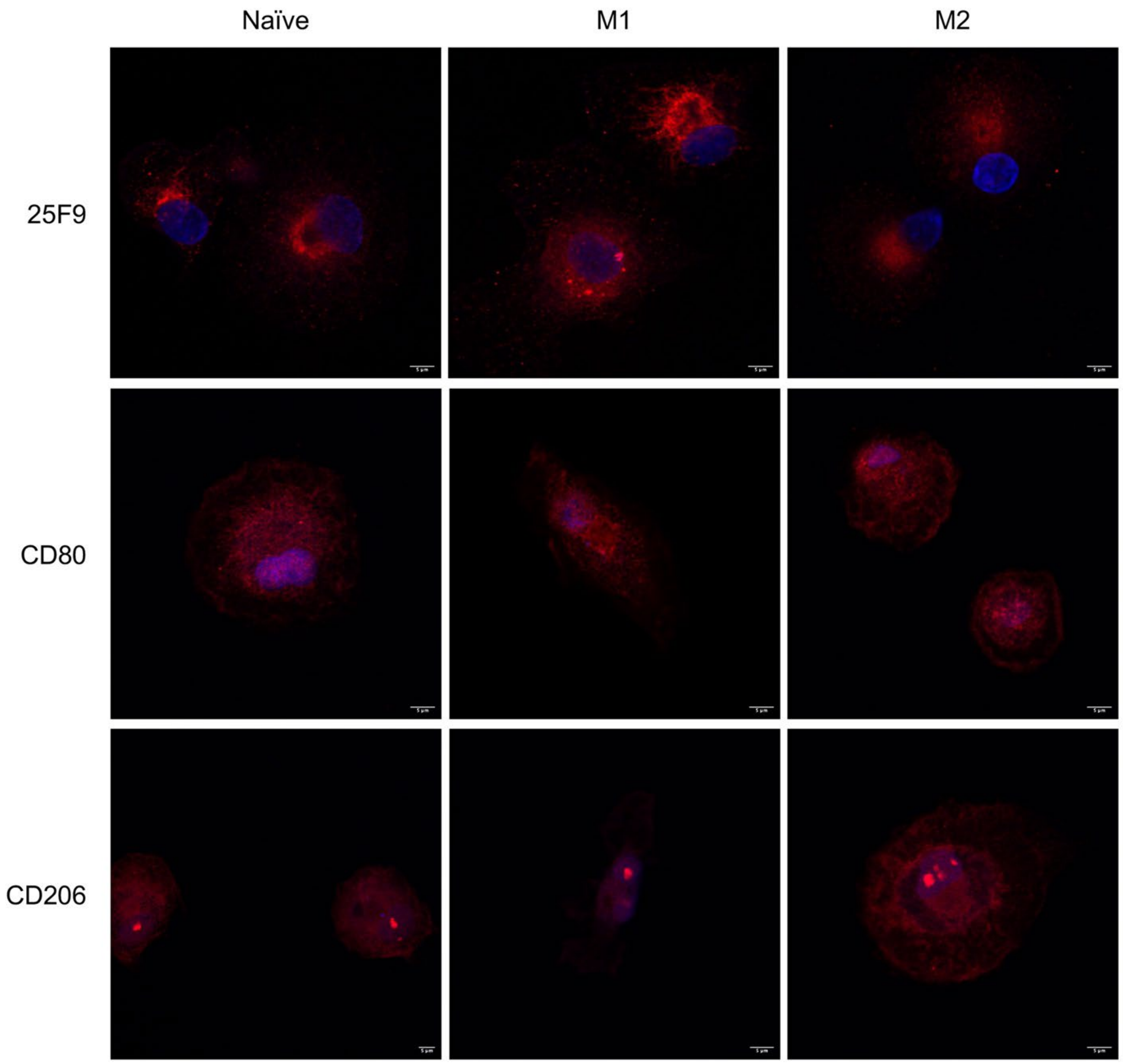

Figure S1. Expression of surface markers specific of mature macrophages in naïve, M1 and M2. 25F29, CD80 and CD206 in red. Cell nuclei are stained blue with Hoechst 33258. Representative images are shown. Bar 5 µm.

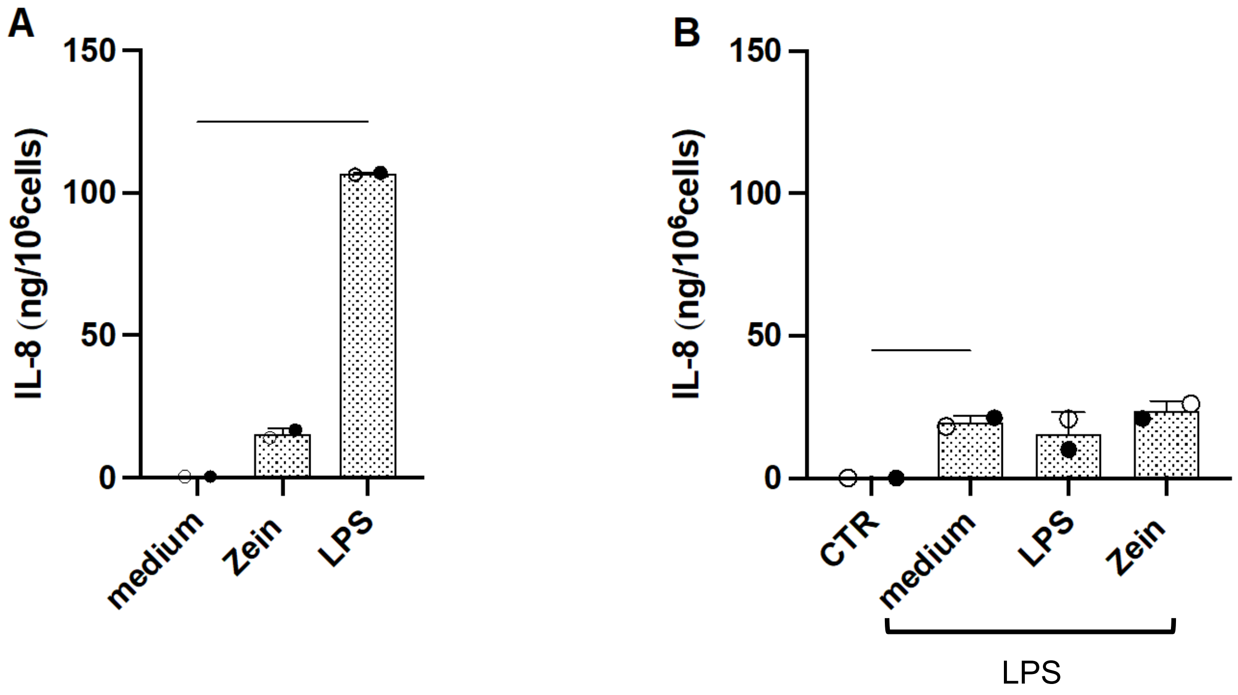

Figure S2. IL-8 production during the primary and secondary innate immune response of human monocytes to zein NPs and LPS. Human blood monocytes were exposed in culture to medium alone or containing zein NPs (100 ng/mL) or LPS (1 ng/mL). The levels of inflammatory IL-8 were measured in supernatants by ELISA after 24 h (A), and after 7 days of resting and re-stimulation for 24 h with medium or with LPS (5 ng/mL) (B). Controls (CTR column) include cells primed with medium, NPs, LPS which are all at baseline after challenge with medium alone. The results from one representative donor are shown. Statistical significance: **** p <0.0001, * p <0.05 in the comparisons between medium vs. LPS and CTR vs. unprimed cells (medium + LPS). All other comparisons are not significant.
